# Semi-Empirical Prediction of Residual Stress Distributions Introduced by Turning Inconel 718 Alloy Based on Lorentz Function

**DOI:** 10.3390/ma13194341

**Published:** 2020-09-29

**Authors:** Huachen Peng, Penghao Dong, Xianqiang Cheng, Chen Zhang, Wencheng Tang, Yan Xing, Xin Zhou

**Affiliations:** 1 School of Mechanical Engineering, Jiulong Lake Campus, Southeast University, Nanjing 211189, China; jsszphuach1992@163.com (H.P.); dongpenghao@outlook.com (P.D.); chengxq_1994@163.com (X.C.); zhangchenseu12138@gmail.com (C.Z.); 2 Shenyang Liming Aero-Engine (Group) Ltd., Shenyang 110000, China; zhou525157569xin@126.com

**Keywords:** semi-empirical prediction, residual stress, finite element model, lorentz function, turning Inconel 718

## Abstract

The residual stress of machined surface has a crucial influence on the performance of parts. It results in large deviations in terms of the position accuracy, dimension accuracy and service life. The purpose of the present study is to provide a novel semi-empirical residual stress prediction approach for turning Inconel 718. In the method, the bimodal Lorentz function was originally applied to express the residual stress distribution. A statistical model between the coefficients of the bimodal Lorentz function and cutting parameters was established by the random forest regression, in order to predict the residual stress distribution along the depth direction. Finally, the turning experiments, electrolytic corrosion peeling, residual stress measurement and correlation analysis were carried out to verify the accuracy of predicted residual stress. The results show that the bimodal Lorentz function has a great fitting accuracy. The adjusted **R^2^** (Ad-**R^2^**) are ranging from 95.4% to 99.4% and 94.7% to 99.6% in circumferential and axial directions, respectively. The maximum and minimum errors of the surface residual tensile stress (SRTS) are 124.564 MPa and 18.082 MPa, those of the peak residual compressive stress (PRCS) are 84.649 MPa and 3.009 MPa and those of the depth of the peak residual compressive stress (DPRCS) are 0.00875 mm and 0.00155 mm, comparing three key feature indicators of predicted and simulated residual stress. The predicted residual stress is highly correlated with the measured residual stress, with correlation coefficients greater than 0.8. In the range of experimental measurement error, the research in the present work provides a quite accurate method for predicting the residual stress in turning Inconel 718, and plays a vital role in controlling the machining deformation of parts.

## 1. Introduction

Nickel-based superalloys, especially Inconel 718, are widely applied in the aviation industry because of their excellent mechanical properties at high temperature [1,2]. However, Inconel 718 is a hard material to be machined even for its wear resistance. In the process of machining, owing to the coupling effect of thermal-mechanical load, complex residual stress distribution will be formed on the machined surface and subsurface, which will affect the machining accuracy, surface integrity and the service life of the parts [3,4,5]. Generally, the residual compressive stress is beneficial to the fatigue life of the parts, while the residual tensile stress is the opposite [6]. The existence of complex residual stress is a severe challenge for the control of dimensional accuracy and shape and position error of the parts. Therefore, it is of great practical significance to study the residual stress distribution of Inconel 718.

At present, the prediction of residual stress mainly focuses on numerical method [7,8,9,10], analytical method [11,12,13,14] and semi-empirical method [15,16,17,18]. In the literature [7,8], the incremental updated Lagrangian method was applied to turning simulations of titanium alloy and nickel alloy, and the predicted residual stress profile has the same shape as the measured in experiment, such as the same hook shape for turning Inconel 718. Mondelin et al. [9] investigated the distribution of thermo-mechanical loads during the simulation of the orthogonal cutting with the Arbitrary Lagrangian and Eulerian (A.L.E.) model. The equivalent loads instead of the thermo-mechanical loads in the cutting process were directly employed to the machined surface without chip deformation and material separation. The predicted stress values are relatively close to the measured. Ulutan et al. [11] established a thermomechanical model to calculate the residual stress, with the shear energy in the primary shear zone, the friction energy in the chip contact zone, and the thermal balance between the chip, tool and workpiece based on the first law of thermodynamics included. This model was further improved in the work of Lazoglu et al. [12]. Liang et al. [13] combined cutting conditions and workpiece material properties into the predictive model for cutting forces, cutting temperatures and residual stresses caused by machining. Furthermore, Huang et al. [14] introduced the heating time of interest points to improve the stress field model on the basis of literature [11,12,13]. The analytical method has an accurate prediction effect for 2D orthogonal cutting.

The numerical method applies the finite element software to simulate the machining process of the material. Given the initial thermal and mechanical boundaries, the residual stress after cutting can be calculated. However, the numerical method needs rich experience in setting the initial thermal and mechanical boundaries, and the simulation process is also computationally expensive. The analytical method mainly is on the basic of thermal equation and elastic-plastic equation to predict the residual stress of the interest point. Compared with the numerical method, the analytical method is time-saving, but the analytical method has made many simplifications in the prediction process, and is only suitable for the prediction of the residual stress in 2D turning [19]. The semi-empirical method utilizes the function fitting simulated or experimental data to obtain the function expression of machining residual stress distribution [20,21,22], which is helpful to study the converse depth of tensile and compressive residual stress and the amplitude and location of the maximum peak compressive residual stress in surface and subsurface.

Polynomial functions [15] and exponentially damped sine/cosine functions [20,21] are often employed in semi-empirical method to fit residual stress data. Ma et al. [15] studied the distribution trend of residual stress by using the six-order polynomials and the influence of the thermal load on the residual stress distribution in the rough machining stage of face turning. In the research of Ulutan et al. [16], exponentially damped cosine function was applied to fit the measured residual stress data of turning and milling nickel-based superalloys. The coefficients of exponentially damped cosine function were determined with particle swarm optimization (PSO) algorithm minimizing the difference between the model and the measurements. Tan et al. [17] utilized an exponential decay function and damped cosine function to study the residual stress distribution after milling, polishing and shot peening. The model coefficients were obtained by establishing regression relationships between the coefficients of the proposed model and the processing parameters. In the literature of Yang et al. [20], a 2D milling simulation finite element model was established, and the finite element results of residual stress distribution along the depth direction were fitted by using the exponentially damped cosine function and particle swarm optimization algorithm. Furthermore, the regression functions between the coefficients of the fitting function and the cutting parameters were applied to predict the distribution of residual stress along the depth direction.

Based on the discussions above, it can be concluded from references [15,16,17,20] that there are fewer undetermined coefficients using the exponentially damped sine/cosine function in the process of fitting residual stress data. However, due to the sine and cosine terms, the fitted subsurface residual stress will fluctuate. Therefore, it is necessary to select a suitable function for fitting the residual stress data to improve the fitting accuracy. Moreover, the main prediction method is to establish the function relationship between the fitting function coefficients and the processing parameters. It is complicated to establish the function relationship and the prediction accuracy needs to be improved.

**Figure 1:**
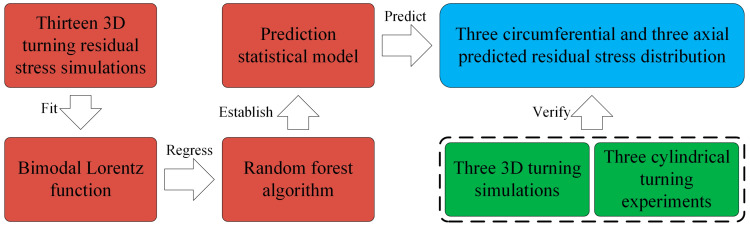
The main steps of residual stress prediction.

Overall, this study aims to develop a novel residual stress model and prediction approach for turning Inconel 718. As shown in Figure 1, thirteen 3D turning simulations are carried out and the residual stress data along the depth direction is extracted. The bimodal Lorentz function is utilized to fit the residual stress distribution for the first time. Simultaneously, the statistical model was established by using random forest method and it realizes the prediction of residual stress distributions under the desired cutting parameters. Compared with three extra simulations and predictions, the three key feature indicators, the surface residual tensile stress (SRTS), the peak residual compressive stress (PRCS) and the depth of peak residual compressive stress (DPRCS), have small error. Furthermore, comparing the prediction model with measured residual stress in experiments, the validity of the prediction model is well verified.

## 2. Methods

### 2.1. FEM Simulation

**Figure 2:**
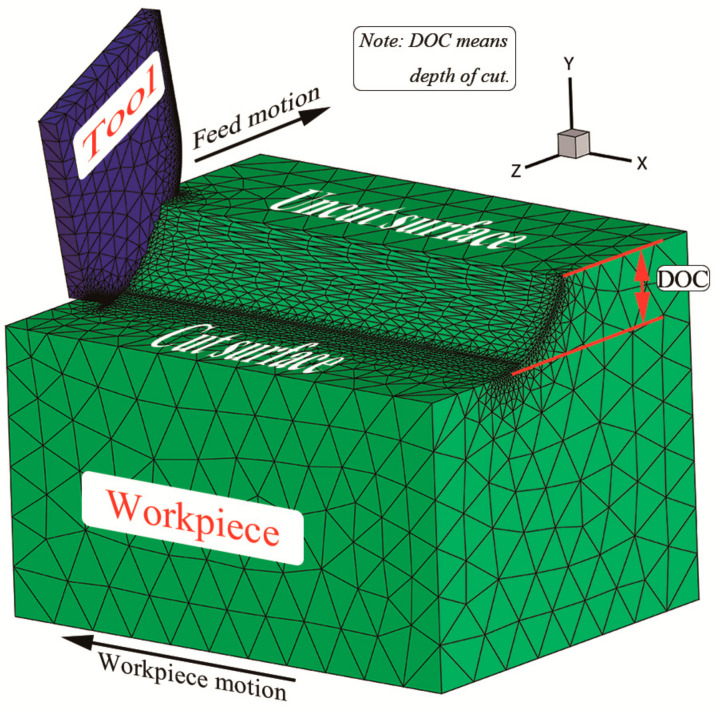
The simplified diagram of 3D finite element turning.

As shown in Figure 2, a 3D turning finite element elastic-plastic model is established by using AdvantEdge V7.4015 software (V7.4015, Third Wave Systems, Minneapolis, MN, USA), in which the workpiece has the dimensions of 5 mm × 3 mm × 2 mm (length × width × height). The DOC means the depth of cut. The AdvantEdge software automatically divides the mesh of the workpiece and the tool with tetrahedral elements. The maximum and minimum element sizes are 0.5 mm and 0.03 mm, respectively. Moreover, in the setting of the workpiece, the adaptive remeshing parameter is 0.005 mm and the curvature-safety keeps 3 so that the finer mesh in the cutting area is automatically divided. And the physical and mechanical properties of Inconel 718 are shown in Table 1, where the Young’s Modulus and Poisson’s Ratio are measured by X-ray diffractometer. The thermal conductivity, specific heat and thermal expansion coefficient are temperature dependent. The Johnson-Cook constitutive model [23] used in the present study is a common constitutive model for researching elastic-plastic materials in Equation (1), including strain hardening effect, strain rate hardening effect and thermal softening effect of materials.

(1) $$\bar{\sigma }\;=\;\underset{Strain\;hardening}{\underset{︸}{\left(A+B{\bar{\varepsilon }}^{n}\right)}}\underset{Strain\;rate\;hardening}{\underset{︸}{\left(1+C\ln\left(\frac{\dot{\bar{\varepsilon }}}{{\dot{\bar{\varepsilon }}}_{0}}\right)\right)}}\underset{Thermal\;softening}{\underset{︸}{\left(1-{\left(\frac{T-T_{room}}{T_{melt}-T_{room}}\right)}^{m}\right)}}$$

where $\bar{\sigma }$ is equivalent plastic stress (MPa), $\bar{\varepsilon }$ is equivalent plastic strain, $\dot{\bar{\varepsilon }}$ is equivalent plastic strain rate ($s^{-1}$), ${\dot{\bar{\varepsilon }}}_{0}$ is reference equivalent plastic strain rate ($s^{-1}$), T is temperature (°C), $T_{melt}$ is melting point of workpiece material (°C), A, B, C, m and n are material parameters. In the preprocessing settings of the simulation, $T_{room}$ was taken as 20 °C. The heat treatment process of Inconel 718 material in the present work includes annealing, solution treatment, primary aging and secondary aging (in Experimental Schedules), which is the similar to the one of the precipitation hardening Inconel 718 in the literature [24]. Thus, A, B, C, m, n and ${\dot{\bar{\varepsilon }}}_{0}$ taken as 1290 MPa, 895 MPa, 0.016, 1.55, 0.526 and 0.03 respectively were applied to the present work.

In addition, the carbide tool with 0.002 mm TiAlN coating was modelled as a rigid body in AdvantEdge software, which has 55° top angle, 1.2 mm nose radius, −7° inclination angle, −6° rake angle, 6° relief angle, −17.5° lead angle and 0.02 mm edge radius. The tool material was set to Carbide-Grade-M. The tool was meshed with the tetrahedral element provided by AdvantEdge software, with maximum element size of 0.3 mm and minimum element size of 0.01 mm and the contact area between the tool, workpiece and chips has a finer mesh automatically. The friction coefficient between the tool and the workpiece is 0.23.

In the simulation, the tool is fixed and the workpiece moves along the cutting direction, that is, the workpiece motion in Figure 2. The length of cut is 6 mm in the setting of cutting parameters. As mentioned above, the length of the workpiece is 5 mm. Therefore, it is the process of 1 mm empty cutting when the workpiece moves along the cutting direction from 5 mm to 6 mm, and the purpose is to make the chips separate from the workpiece and realize the complete machining of the workpiece.

**Figure 3:**
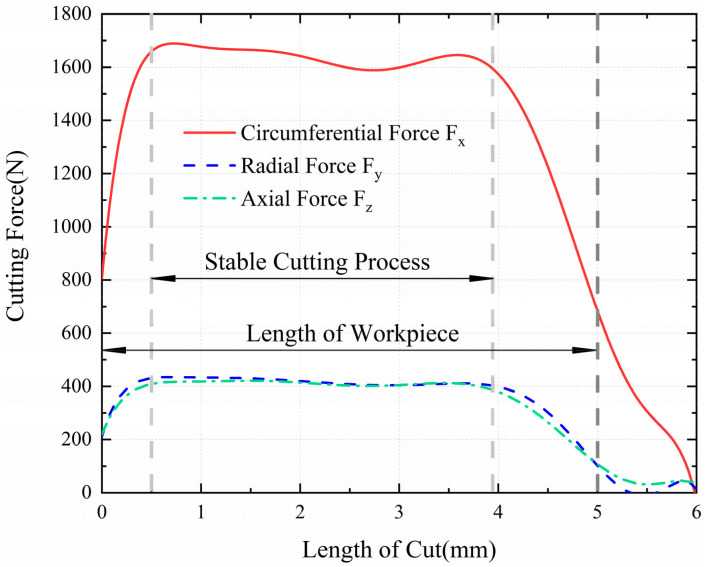
The cutting forces fitting diagram of simulation process.

**Figure 4:**
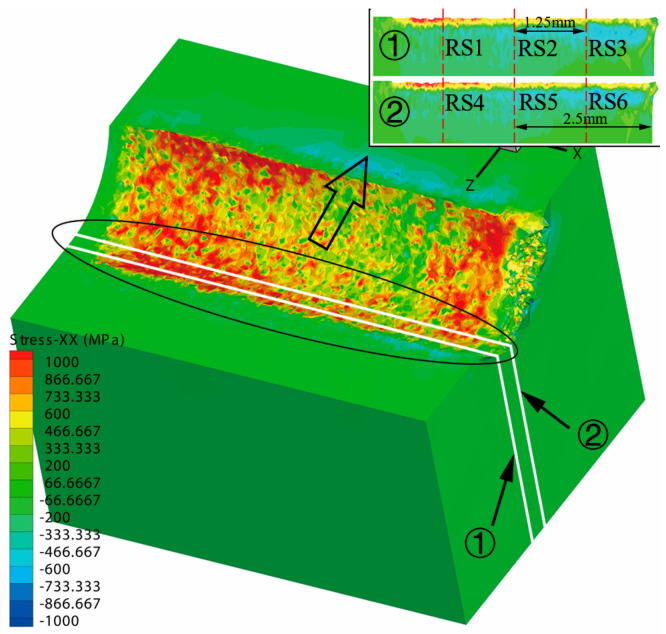
The schematic diagram of residual stress extraction.

According to the change of cutting forces in the whole cutting process, the Figure 3 illustrates that the cutting forces are stable between 0.5 mm and approximately 4 mm, and the fluctuation of cutting forces is gradual. The research on turning residual stress in this range is closer to the actual situation of cylindrical turning. The residual stress distribution in the circumferential direction (X direction) in Figure 4 is not uniform. The mechanical load and thermal load are coupled with each other during the machining process. When the tool starts to cut into the workpiece, the heat dissipation conditions of the tool, workpiece and chips are conducive to heat dissipation. As the cutting continues, the heat dissipation capacity becomes weaker, the thermal softening effect of the material is enhanced, and the cutting force is reduced (approximately 3 mm in Figure 3). The strength of this effect is related to feed, depth of cut and cutting speed, which causes the different degrees of non-uniformity in stress distribution. The literature [26] has given the method to extract the residual stress. In the simulation results, two planes (① and ② in Figure 4) were sliced, and the residual stress data were extracted at 1.25 mm, 2.5 mm and 3.75 mm of these two planes respectively, which are recorded as RS1, RS2, RS3, RS4, RS5 and RS6, as shown in Figure 4. The three distances above are all in the range of 0.5 mm to 4 mm, which belong to the stable cutting process. In order to reduce the simulation error and non-uniformity of the residual stress, the residual stress to be studied is the average value of 6 groups of the extracted data, namely:

(2) $$\sigma \left(h\right)\;=\;\frac{1}{6}\sum_{i=1}^{6}RSi$$

where *h* is the depth along the radial direction from the cutting surface (mm); $\sigma \left(h\right)$ is the residual stress along the radial direction (MPa); RS is the abbreviation of the residual stress.

### 2.2. Random Forest Regression

**Figure 5:**
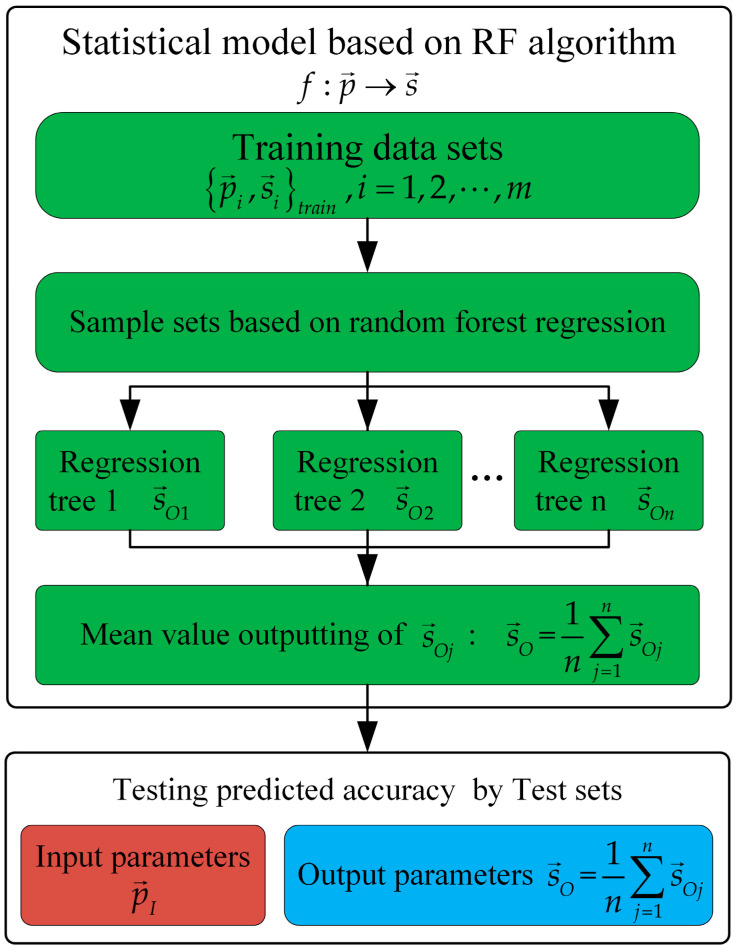
The parameters prediction flow chart of random forest regression.

The decision trees and regression methods are the approaches to establish predictive models [27,28]. In the literature [29], a prediction model of ore crushing plate lifetimes was proposed based on the decision trees and artificial neural networks. Random forests are an effective tool in prediction. On the basis of bagging algorithm [30], some sample features are randomly selected from all the sample features, in which an optimal sample feature is chosen as the sub partition of the decision tree on the root node. For regression problems, the final prediction is the average value from the prediction of all the trees in the prediction sets. In this way, the bagging predictors is improved by random forests to ensure the accuracy of prediction [31]. In the present work, the random forest algorithm [18] realizes the prediction between cutting parameters and residual stress distribution. As shown in Figure 5, the simulation results under the known cutting parameters $\vec{p}$ are fitted to obtain the independent parameters $\vec{s}$ of the corresponding fitting function, which can get the training data sets ${\left\{{\vec{p}}_{i},{\vec{s}}_{i}\right\}}_{train},i=1,2,\cdot \cdot \cdot ,m$ (m is the number of training data sets.). However, in order to predict the parameters ${\vec{s}}_{O}$ of the residual stress fitting function corresponding to the input cutting parameters ${\vec{p}}_{I}$, it is necessary to establish the mapping relationship $f:\vec{p}\to \vec{s}$ between the cutting parameters $\vec{p}$ and the parameters $\vec{s}$ of the function. Generally, equation $\vec{s}=f\left(\vec{p}\right)$ expressed by specific function is applied to establish the mapping relationship. However, it is difficult to give the specific functional relationship because of the complexity between the residual stress distribution and the cutting parameters.

By comparison, the random forest algorithm is able to establish the mapping relationship $f:\vec{p}\to \vec{s}$ between $\vec{p}$ and $\vec{s}$ without giving the specific equation by utilizing the bagging method to carry out the random sampling with return of the training data sets and applying the regression tree to the fitting of the corresponding random samples. Test sets are applied to test the predicted accuracy (in Results and Discussions). For the input parameters ${\vec{p}}_{I}$ in the test sets, the random forest algorithm gives the output value ${\vec{s}}_{Oj}$ of each regression tree, and takes the mean value of ${\vec{s}}_{Oj}$ as the final predicted value ${\vec{s}}_{O}$.

### 2.3. Model of Residual Stress Based on Lorentz Function

**Figure 6:**
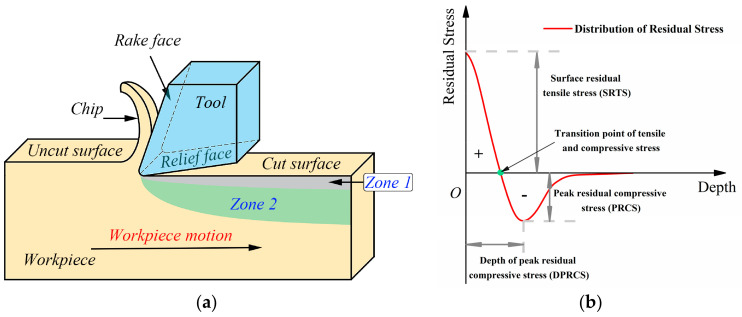
(**a**) The simplified diagram of turning process; (**b**) The typical distribution of residual stress along the depth direction.

As is known to all, the mechanical load generally causes the residual compressive stress, and the thermal load often produces the residual tensile stress. As shown in Figure 6a, the Zone 1 is near the machining surface and the Zone 2 is far from the surface. The mechanical load leads to plastic deformation of materials in the Zone 1, while the materials have elastic strain in the Zone 2. With the mechanical load removed, the materials in the Zone 1 still retain large plastic strain, while the strain in the Zone 2 remains at a low level. Therefore, the materials in the Zone 1 form residual compressive stress under the constraint of materials in the Zone 2. In contrast, the thermal gradient in the Zone 1 is larger than that in the Zone 2, so the materials in the Zone 1 keep a larger thermal expansion than that in the Zone 2. After cooling, residual tensile stress is formed in the Zone 1 due to the limitation of the inner layer materials in the Zone 2. Therefore, the coupled mechanical and thermal loads result in the residual stress profile in the machined surface layer of the parts. The effect of thermal load is more obvious than that of mechanical load on the machined surface of Inconel 718 material, so the residual tensile stress state is formed on the surface. With the increase of depth, the effect of mechanical load on the inner layer material is enhanced, which make it change to the residual compressive stress state, and there is a peak value of the residual compressive stress. Finally, the residual stress remains at the level in the bulk material. The three key feature indicators of residual stress distribution along the depth direction are shown in Figure 6b, including the SRTS, the PRCS and the DPRCS. The distribution trend of residual stress along the depth direction can be expressed by a proper function. The random forest algorithm predicts the residual stress distribution in the turning Inconel 718 material under the desired cutting parameters.

The Lorentz function, applied to fit spectral characteristics, has an excellent effect for fitting data with peak characteristics. Equation (3) is one of the expressions of the Lorentz function. On the basis of the Lorentz function, Equation (4), called the bimodal Lorentz model, was proposed to predict the residual stress distribution along the depth direction of the surface layer after turning Inconel 718. In Equation (4), *h* is the independent variable and $\sigma \left(h\right)$ is the dependent variable. There are five undetermined coefficients, namely $\sigma_{0}$, $A_{1}$, $A_{2}$, ω and $h_{c}$.

(3) $$L\left(x\right)=\frac{2}{\pi }\times \frac{\omega }{4{\left(x-x_{c}\right)}^{2}+\omega^{2}}$$

(4) $$\sigma \left(h\right)=\sigma_{0}+\frac{2A_{1}}{\pi }\times \frac{0.2}{4h^{2}+0.04}+\frac{2A_{2}}{\pi }\times \frac{\omega }{4{\left(h-h_{c}\right)}^{2}+\omega^{2}}$$

For the continuous function on the interval [a, b]:

(5) $$y=f\left(x\right)$$

There is the extreme point $x_{e}$ and corresponding extreme value $y_{e}$, satisfying the condition of Equation (6).

(6) $$\left\{\begin{array}{l} {\frac{dy}{dx}}_{|x=x_{e}}={\frac{df\left(x\right)}{dx}}_{|x=x_{e}}=0\\ y_{e}=f\left(x_{e}\right) \end{array}\right.$$

Similarly, the extreme point $h_{e}$ and corresponding extreme value $\sigma_{e}$ of the Equation (4) satisfy the Equation (7):

(7) $$\left\{\begin{array}{l} {\frac{d\sigma \left(h\right)}{dh}}_{\left|h=h_{e}\right.}=0\\ \sigma_{e}=\sigma \left(h_{e}\right) \end{array}\right.$$

Therefore, in Equation (4), the extreme point ${\left\{h_{e}\right\}}_{train}$ of the residual stress distribution under each group of cutting parameters ${\left\{v_{c},a_{p},f\right\}}_{train}$ in the simulation data set was calculated by the derivative of $\sigma \left(h\right)$, and the extreme value ${\left\{\sigma_{e}\right\}}_{train}$ of the residual stress distribution was further obtained. In this way, the key parameters ${\left\{A_{1},A_{2},\omega ,h_{c},\sigma_{e}\right\}}_{train}$ were adopted to determine fitting function rather than five coefficients, and the random forest regression was utilized to establish the corresponding relationship between cutting parameter ${\left\{v_{c},a_{p},f\right\}}_{train}$ and key parameters ${\left\{A_{1},A_{2},\omega ,h_{c},\sigma_{e}\right\}}_{train}$, so as to further predict the residual stress distribution under the desired cutting parameters ${\left\{v_{c},a_{p},f\right\}}_{desired}$ (cutting parameters in test sets). That is to say, five parameters ${\left\{A_{1},A_{2},\omega ,h_{c},\sigma_{e}\right\}}_{predict}$ predicted by random forest regression determine the residual stress distribution equation under the desired cutting parameters ${\left\{v_{c},a_{p},f\right\}}_{desired}$.

## 3. Experimental Schedules

### 3.1. Workpiece and Cutting Tool

Inconel 718 pipes were used in turning experiments, with outer diameter of 76 mm, wall thickness of 8 mm and axial length of 200 mm. After annealed, 720 °C/8 h, cooled at 50 °C/h to 620 °C, held at 620 °C/8 h and quick cooled, Inconel 718 has the hardness of 43 HRC. The chemical composition of the material is shown in Table 2.

**Figure 7:**
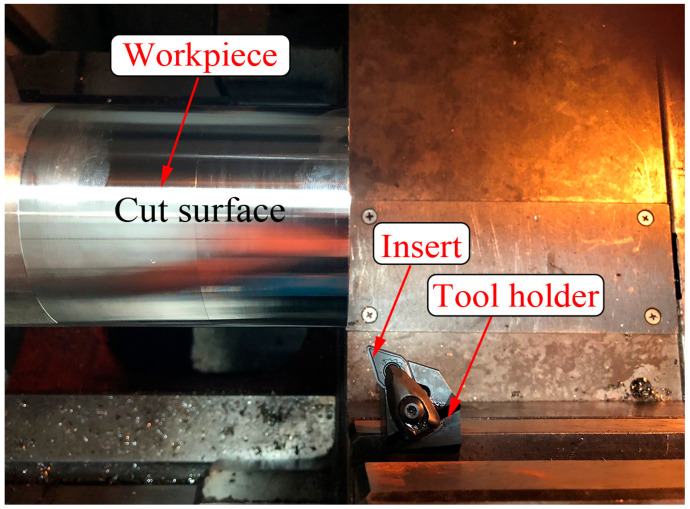
The experimental details of turning Inconel 718 pipes.

The turning experiments were carried out on the SK50P horizontal CNC machine tool. In Figure 7, the tool holder (DDHNR 2525M 1504) has the section of 25 mm × 25 mm and the inclination angle of −7°. Insert (DNMG 15 04 12-SMR 1105) is a D-type (55° top angle) tool with PVD (TiAlN) coating and tool nose radius of 1.1906 mm. Other parameters are consistent with the tool settings in the simulation. Moreover, each test is dry cut with a new insert.

### 3.2. Turning Parameters

There are 16 sets of cutting parameters using 3-factor 4-level orthogonal table, namely *L*_16_(4^3^). Among them, 13 sets of parameters were simulated to establish the statistical model of residual stress. The turning experiments adopted the remaining three sets of cutting parameters in Table 3. In Section 4.3, the results of turning experiments will be applied to verify the residual stress prediction model.

### 3.3. Measurements of Residual Stress

**Figure 8:**
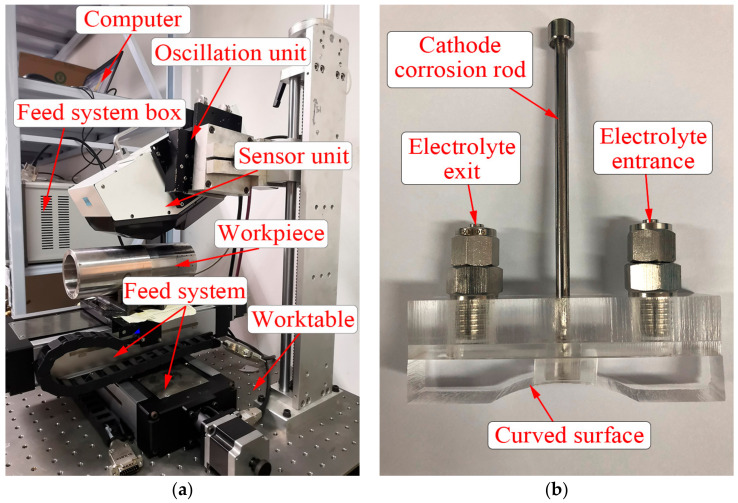
(**a**) The details of X-ray residual stress measurement; (**b**) The electrolytic corrosion device.

In the experiments, the μ—360n type X-ray diffractometer was utilized to measure the surface residual stress, shown in Figure 8a. The residual stress of the cut surface can be measured by X-ray diffraction without damaging the cutting surface, which is one of the reliable methods to obtain the residual stress of the machined surface. In the measurement, Cr K-Beta tube was applied with 30 kV voltage and 1.2 mA current, the X-ray wavelength was 2.08480 A, 311 crystal plane was selected, and diffraction angle (2Theta) and diffraction lattice angle were 150.876° and 29.124° respectively.

The points to be measured were calibrated on the cut surface of the workpiece after turning. Then, the workpiece was placed on the base of the feeding system worktable. The height of workpiece was adjusted with the lead screw, which made the spot of the X-ray diffractometer coincide with the measured point on the cut surface by the feed system control box. During the measurement, the oscillation unit continuously adjusted the angle, so that the sensor unit could measure the Inconel 718 material at the right angle of measurement, and the residual stress values along the circumferential and axial directions of the machined surface were obtained by computer. Furthermore, the electrolytic corrosion method was adopted in order to study the trend of the surface residual stress along the depth direction after turning Inconel 718 material. Compared with chemical corrosion, the electrolytic corrosion method has higher efficiency and controllable corrosion depth. Figure 8b shows the electrolytic corrosion device, in which the curved surface fits the machined surface of the Inconel 718 pipes. The electrolyte enters from the electronic entrance through the peristaltic pump, flows through the cathode corrosion rod and the workpiece surface, and finally flows into the electrolytic cell from the electronic exit, where the workpiece is the anode. The residual stress values at different depths were obtained by electrolytic corrosion peeling and the residual stress measurement after turning Inconel 718 pipes. Table 4 shows the electrolytic corrosion parameters in the peeling.

## 4. Results and Discussions

### 4.1. Statistical Model of Predicting Residual Stress

For 3-factor 4-level orthogonal table *L*_16_(4^3^), except for three sets of cutting parameters used in experimental verification, the remaining 13 groups of cutting parameters were simulated by finite element method and then residual stress data were extracted. The Equation (2) calculated the average value of residual stress in circumferential and axial directions, and moreover the five key parameters ${\left\{A_{1},A_{2},\omega ,h_{c},\sigma_{e}\right\}}_{train}$ were obtained by using Equation (4) to fit the data of the simulation. Then, the random forest algorithm established the statistical model of predicting residual stress. Table 5 and Table 6 show the key parameter values of the fitting function of circumferential and axial residual stress respectively, where the closer the Ad-**R^2^** is to 1, the higher the fitting accuracy is. As shown in Table 5 and Table 6, the Ad-**R^2^** is not less than 0.947, indicating that the fitting effect of residual stress with Equation (4) is desirable.

The random forest algorithm was applied to establish the mapping relationship between cutting parameters ${\left\{v_{c},a_{p},f\right\}}_{train}$ and fitting function key parameters ${\left\{A_{1},A_{2},\omega ,h_{c},\sigma_{e}\right\}}_{train}$, and predict the residual stress profile under the desired cutting parameters on the basic of the training data sets, i.e., the cutting parameters and the key parameters in Table 5 and Table 6. In the literature [32], the **R^2^** values of the test sets are in the range of 0.797 and 0.890. Therefore, in the present study, **R^2^** values were controlled between 0.80 and 0.85 to avoid over fitting. Therefore, the statistical model takes the cutting parameters in Table 3 as the input parameters, and Table 7 shows the predicted five key parameters corresponding to the bimodal Lorentz function (Equation (4)).

### 4.2. Residual Stress Comparisons between Simulation and Prediction

**Figure 9:**
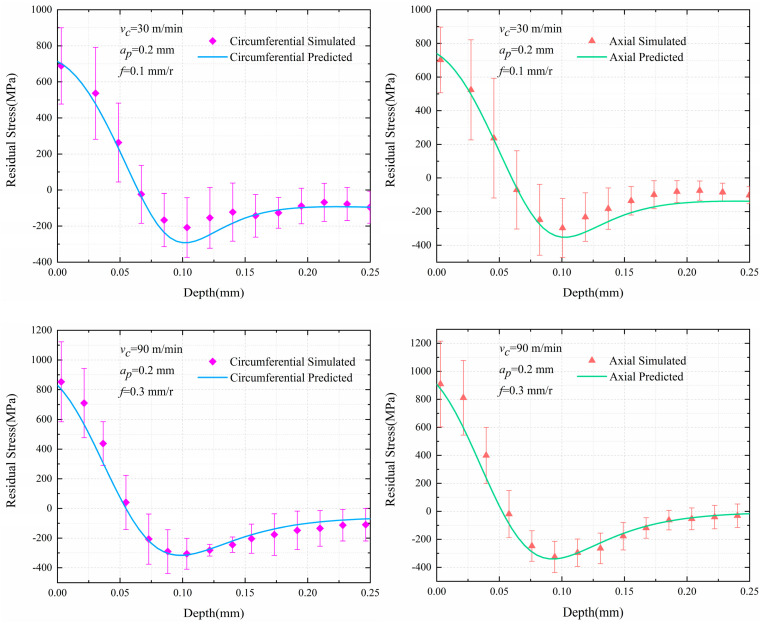

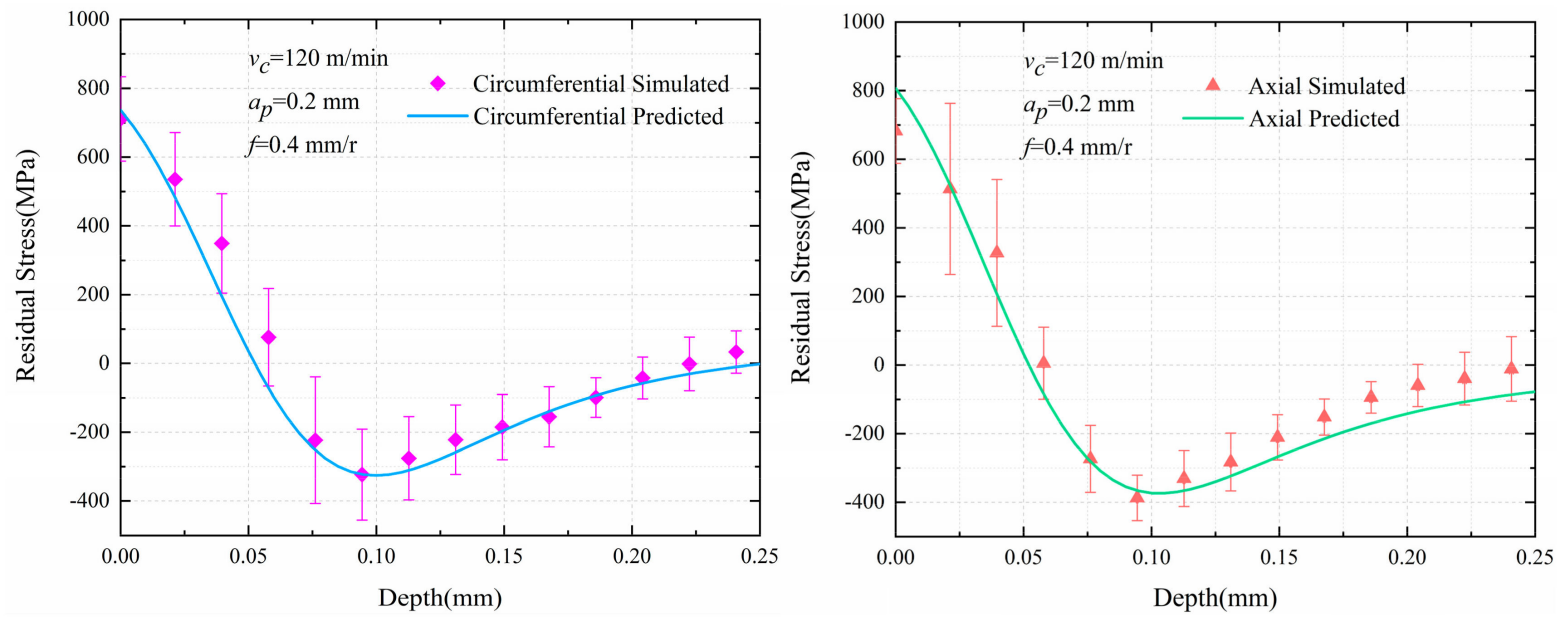
The comparisons of simulated and predicted residual stress results.

**Figure 10:**
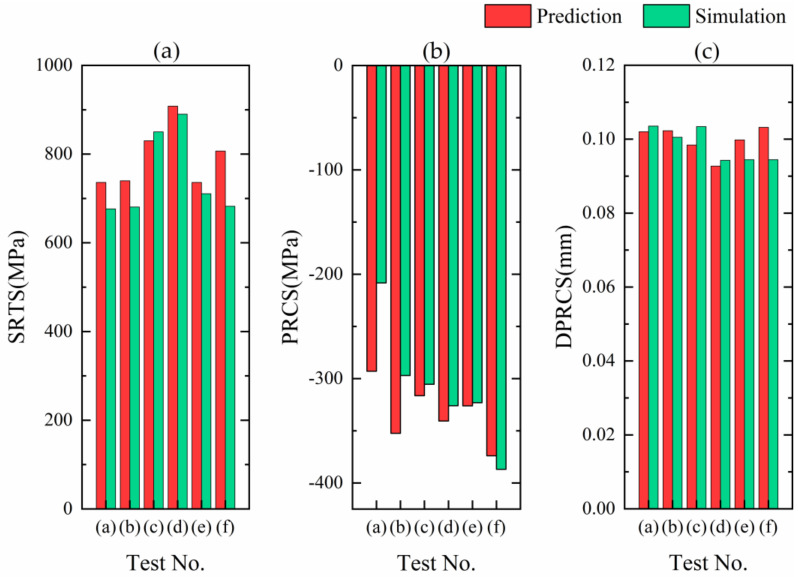
The indicators of predicted and simulated residual stress distribution. (**a**) The comparisons of predicted and simulated SRTS; (**b**) The comparisons of predicted and simulated PRCS; (**c**) The comparisons of predicted and simulated DPRCS.

Three sets of cutting parameters in Table 7 were simulated, and the simulation data was compared with the predicted results. Figure 9 shows the comparisons between the predicted residual stress distribution curves and the simulation results. It indicates that the curves predicted by the three sets of cutting parameters, whether circumferential residual stress or axial residual stress, have good consistency with the simulation results. The three key feature indicators, SRTS, PRCS and DPRCS, of six residual stress distributions along the depth direction in Table 7 are compared, as shown in Figure 10. It can be indicated that for the SRTS, the difference between the predicted value and the simulation result of test (f) is the largest, the error is 124.564 MPa, and that of test (d) is the smallest, the error is 18.082 MPa, in Figure 10a; besides, Figure 10b shows that for the PRCS, the difference between the predicted value and the simulation result of test (a) is the largest, with an error of 84.649 MPa, and that of test (e) is the smallest, with an error of 3.009 MPa; moreover, Figure 10c illustrates that for the DPRCS, the difference between the predicted value and the simulation result of test (f) is the largest, the error is 0.00875 mm, and that of test (a) is the smallest, the error is 0.00155 mm.

The prediction of residual stress distribution is in good agreement with the simulation results for turning Inconel 718 pipes. Therefore, it is proved that the Equation (4) and the random forest algorithm are acceptable for prediction of residual stress distribution in turning Inconel 718 pipes.

### 4.3. Experimental Verification

**Figure 11:**
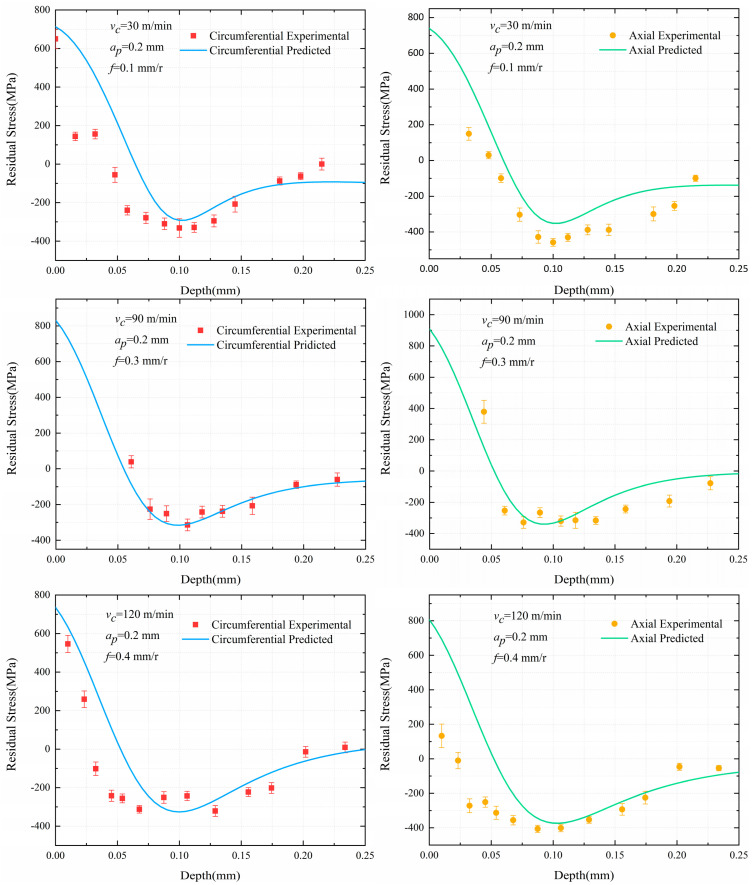
The comparisons of measured and predicted residual stress results.

The turning experiments were carried out to prove that Equation (4) is reliable for predicting the residual stress distribution of Inconel 718 material. The predicted curves were compared with the residual stress data measured in the experiments. Figure 11 indicates that the experimental data and prediction curves have good consistency. Besides, the stress values of points were calculated on each prediction curve with abscissas of the points consistent with the abscissas of the experimental measured points. Therefore, two groups of point sets were got, $\left\{\left(h_{E},\sigma_{E}\right)\right\}$ and $\left\{\left(h_{E},\sigma_{p}\right)\right\}$. Based on the correlation analysis on $\left(\sigma_{E},\sigma_{p}\right)$, Table 8 shows correlation coefficients between the experimental stress values and the predicted stress values, calculated by Equation (8).

(8) $$r=\frac{\sum_{i=1}^{n}\left(\sigma_{Ei}-{\bar{\sigma }}_{E}\right)\left(\sigma_{pi}-{\bar{\sigma }}_{p}\right)}{\sqrt{\sum_{i=1}^{n}{\left(\sigma_{Ei}-{\bar{\sigma }}_{E}\right)}^{2}}\sqrt{\sum_{i=1}^{n}{\left(\sigma_{pi}-{\bar{\sigma }}_{p}\right)}^{2}}}$$

where $\sigma_{E}{}_{i}$ represents the *i*th measured residual stress value, ${\bar{\sigma }}_{E}$ represents the average value of the measured residual stress, $\sigma_{pi}$ represents the *i*th predicted residual stress value, and ${\bar{\sigma }}_{p}$ represents the average value of the predicted residual stress, all in MPa.

**Figure 12:**
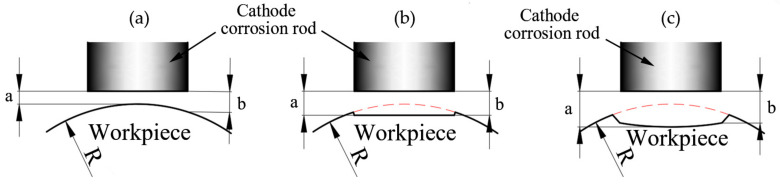
The schematic diagram of electrolytic corrosion process. (**a**) The distance between the center of the cathode corrosion rod and the workpiece is less than that between the edge of the cathode corrosion rod and the workpiece, a < b; (**b**) a = b; (**c**) a > b.

**Figure 13:**
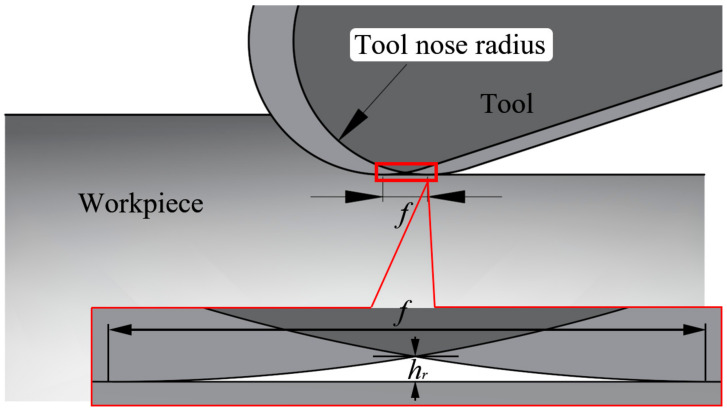
The schematic diagram of cutting residual height.

In Figure 11, the predicted curves and the experimental measured distributions have the same hook shape, but the predicted curves are higher than the experimental residual stress values before the depth of 0.05mm in most cases. The depth was calculated by the rate and time of electrolytic polishing in the experiment. At the beginning of the electrolytic process, the top of the workpiece is closer to the cathode corrosion rod (a < b), so the corrosion rate is faster than the surrounding, as shown in Figure 12a. This effect is obvious at the initial stage of corrosion and will gradually weaken with the progress of electrolytic corrosion, as illustrated in Figure 12b,c. Therefore, in the initial stage of corrosion, the actual depth of corrosion is greater than that obtained by the product of polishing rate and time (depth value in Figure 11), which causes the measured residual stress to be less than the predicted before the depth of 0.05 mm. In particular, there is a slight increase in circumferential residual stress (***v_c_*** = 120 m/min, ***a_p_*** = 0.2 mm, ***f*** = 0.4 mm/r), which is due to the influence of cutting residual height on electrolytic corrosion depth. In Figure 13 and Equation (9), the residual height increases with the increase of feed. The existence of residual height increases the error between actual depth and timing depth, which makes the maximum residual compressive stress peak appear in advance. This effect is more significant with the increase of feed. Therefore, the circumferential and axial residual stresses measured in the experiment shifted slightly to the left in Figure 11 for ***v_c_*** = 120 m/min, ***a_p_*** = 0.2 mm, ***f*** = 0.4 mm/r, resulting in a slight increase in the circumferential direction.

(9) $$h_{r}=R_{tool-nose}-\sqrt{R_{tool-nose}^{2}-{\left(\frac{f}{2}\right)}^{2}}$$

where $h_{r}$ is the cutting residual height, $R_{tool-nose}$ is the tool nose radius and *f* is the feed rate.

In the Test No. (a) to (f), the correlation coefficients between the measured and predicted results are 0.8776, 0.9588, 0.9193, 0.8702, 0.8558 and 0.8180 respectively. It is found that the correlation coefficients between the predicted values and the experimental data of six groups of tests are between 0.8 and 1.0 in Table 8, indicating that the predicted results are highly correlated with the experimental data. The validity of the prediction model is well verified considering the experimental measurement error and the bimodal Lorentz model is acceptable.

## 5. Conclusions

The residual stress of cylindrical turning Inconel 718 pipe was studied in this paper. A new prediction equation of residual stress distribution was proposed on the basic of the Lorentz function. Additionally, comparing the results of prediction and simulation, and comparing the data of prediction and experiment, the results show that the prediction model of residual stress is accurate for prediction of residual stress distribution of turning Inconel 718 material by using random forest regression. According to the research content, it is summarized as follows:

1. The bimodal Lorentz model (Equation (4)) has a good accuracy for fitting the residual stress distribution, whose Ad-**R^2^** for fitting simulated residual stress data is over 0.947. The statistical model of residual stress prediction is established by random forest regression based on the training data sets, and the precision of the testing sets is controlled between 0.80 and 0.85. The key parameters $\left\{A_{1},A_{2},\omega ,h_{c},\sigma_{e}\right\}$ of the prediction model are predicted under the expected cutting parameters in the testing sets.
2. The three extra simulations and experiments are carried out under the desired cutting parameters in the testing sets, and the three key feature indicators are compared between the simulated and predicted residual stress, including the SRTS, the PRCS, and the DPRCS. The results show that the maximum and minimum values of the SRTS error are 124.564 MPa and 18.082 MPa respectively, those of the PRCS error are 84.649 MPa and 3.009 MPa respectively, and those of the DPRCS error are 0.00875 mm and 0.00155 mm respectively.
3. The distributions of predicted residual stress and experimental data show the hook shape. The residual stress predicted by the random forest regression is over predicted before the depth of 0.05 mm, that is, the predicted values are greater than the measured values in the experiment. This is due to the error between the product of the polishing rate and time and the actual depth in the electrolytic cylindrical surface.
4. The correlation analysis was carried out between the measured and predicted residual stress based on the experiments and the statistical model of random forest regression and the result shows that the correlation coefficient is between 0.8 and 1.0, indicating that they are highly correlated.

On the basic of the present work, the accuracy of electrolytic corrosion depth needs to be improved in the following work, and further research can be conducted on the real-time control of the stress level during the cutting process, the improvement of the process planning and the deformation control of the components.

## Figures and Tables

**Table 1:** Physical and mechanical properties of Inconel 718 [25].

Density (kg/m^3^)	Young’s Modulus (GPa)	Poisson’s Ratio	Thermal Conductivity (W/(M·K))	Specific Heat (J/(kg·K))	Thermal Expansion Coefficient (10^−6^/K)	Melting Temperature (K)
8240	214.58	0.305	10.53 (293 K)	435 (293 K)	11.8 (293 K–373 K)	1573
			14.7 (373 K)	481.4 (573 K)	13 (293 K–573 K)	
			17.8 (573 K)	514.8 (773 K)	14.1 (293 K–673 K)	
			19.6 (773 K)	573.4 (973 K)	14.8 (573 K–873 K)	

**Table 2:** The composition of main elements in Inconel 718.

Elements	Ni	Fe	Cr	Nb	Mo	Ti
Weight (%)	52.860	19.150	19.085	5.085	3.105	0.710

**Table 3:** The turning experiment parameters.

No. of Experiments	Cutting Speed *v_c_* (m/min)	Feed Rate *f* (mm/r)	Depth of Cut *a_p_* (mm)
1	30	0.1	0.2
2	90	0.3	0.2
3	120	0.4	0.2

**Table 4:** The electrolytic parameters.

Electrolytic Parameters	Values
Electrolyte	10% NaCl
Electrolyte speed	800 mL/min
Voltage	24 V
Electric current	3 A
Polishing rate	0.005 mm/s

**Table 5:** The key parameters of circumferential residual stresses fitting function (***v_c_***: cutting speed in m/min; ***f***: feed rate in mm/r; ***a_p_***: depth of cut in mm).

No.	*v_c_*	*f*	*a_p_*	*A*_1_	*A*_2_	*ω*	*h_c_*	*σ_e_*	Ad-R^2^
1	30	0.2	0.4	364.017	−104.180	0.099	0.0896	−285.156	0.979
2	30	0.3	0.6	263.666	−106.190	0.120	0.0976	−333.720	0.952
3	30	0.4	0.8	547.769	−454.569	0.235	0.0769	−451.186	0.994
4	60	0.1	0.4	444.092	−214.326	0.142	0.0774	−352.967	0.991
5	60	0.2	0.2	252.347	−111.825	0.112	0.0811	−288.342	0.972
6	60	0.3	0.8	891.701	−725.995	0.212	0.0428	−377.403	0.982
7	60	0.4	0.6	460.210	−287.150	0.172	0.0807	−424.856	0.981
8	90	0.1	0.6	346.455	−169.498	0.124	0.0852	−378.482	0.971
9	90	0.2	0.8	470.750	−292.847	0.159	0.0711	−525.055	0.984
10	90	0.4	0.4	616.592	−492.188	0.213	0.0633	−379.655	0.980
11	120	0.1	0.8	355.264	−180.584	0.137	0.0854	−375.208	0.972
12	120	0.2	0.6	503.378	−307.003	0.148	0.0663	−421.425	0.986
13	120	0.3	0.4	388.973	−216.087	0.147	0.0745	−320.907	0.988

**Table 6:** The key parameters of axial residual stresses fitting function (***v_c_***: cutting speed in m/min; ***f***: feed rate in mm/r; ***a_p_***: depth of cut in mm.).

No.	*v_c_*	*f*	*a_p_*	*A*_1_	*A*_2_	*ω*	*h_c_*	*σ_e_*	Ad-R^2^
1	30	0.2	0.4	435.127	−174.628	0.111	0.0852	−463.574	0.985
2	30	0.3	0.6	323.190	−137.632	0.127	0.0952	−342.327	0.947
3	30	0.4	0.8	852.431	−702.291	0.238	0.0543	−401.623	0.992
4	60	0.1	0.4	471.094	−269.212	0.148	0.0754	−393.947	0.990
5	60	0.2	0.2	368.356	−210.993	0.137	0.0733	−324.781	0.980
6	60	0.3	0.8	649.120	−430.848	0.177	0.0636	−401.583	0.990
7	60	0.4	0.6	564.902	−368.620	0.182	0.0746	−383.094	0.980
8	90	0.1	0.6	396.488	−233.340	0.134	0.0783	−468.865	0.979
9	90	0.2	0.8	480.306	−290.734	0.151	0.0730	−406.263	0.992
10	90	0.4	0.4	682.622	−506.349	0.202	0.0593	−331.225	0.987
11	120	0.1	0.8	515.575	−349.894	0.175	0.0775	−420.300	0.991
12	120	0.2	0.6	590.375	−432.663	0.169	0.0618	−528.207	0.996
13	120	0.3	0.4	577.711	−372.320	0.171	0.0664	−347.409	0.992

**Table 7:** The predicted key parameters of the residual stress prediction function (***v_c_***: cutting speed in m/min; ***f***: feed rate in mm/r; ***a_p_***: depth of cut in mm).

v_c_	f	a_p_	A_1_	A_2_	ω	h_c_	σ_e_	Direction	Test No.
30	0.1	0.2	343.879	−128.140	0.116	0.0875	−292.893	Circumferential	(a)
			389.361	−163.529	0.128	0.0846	−352.447	Axial	(b)
90	0.3	0.2	511.643	−308.741	0.165	0.0682	−316.324	Circumferential	(c)
			557.601	−347.867	0.159	0.0660	−340.623	Axial	(d)
120	0.4	0.2	475.457	−369.108	0.185	0.0677	−326.093	Circumferential	(e)
			575.243	−438.225	0.194	0.0641	−373.932	Axial	(f)

**Table 8:** The correlation coefficients between measured and predicted results.

Test No.	(a)	(b)	(c)	(d)	(e)	(f)
*r*	0.8776	0.9588	0.9193	0.8702	0.8558	0.8180
